# Identification and Quantification of the Major Phenolic Constituents in *Castanea sativa* and Commercial Interspecific Hybrids (*C. sativa* x *C. crenata*) Chestnuts Using HPLC–MS/MS

**DOI:** 10.3390/ijms241713086

**Published:** 2023-08-23

**Authors:** Aljaz Medic, Petra Kunc, Tilen Zamljen, Metka Hudina, Robert Veberic, Anita Solar

**Affiliations:** Biotechnical Faculty, Department of Agronomy, University of Ljubljana, Jamnikarjeva 101, SI-1000 Ljubljana, Slovenia; petra.kunc@bf.uni-lj.si (P.K.); metka.hudina@bf.uni-lj.si (M.H.);

**Keywords:** phenolic compounds, HPLC-MS, dicarboxylic acid, wild accessions, chestnut

## Abstract

Due to the lack of studies on chestnut metabolites, this study was conducted to identify and quantify the major phenolic constituents in chestnuts. Data were compared with the three most commonly grown interspecific hybrids of *C. sativa* and *C. crenata* (‘Bouche de Betizac’, ‘Marsol’, and ‘Maraval’) and three “native” accessions of *C. sativa*. High-performance liquid chromatography coupled with mass spectrometry was used to identify and quantify these compounds. Four dicarboxylic acid derivatives, five hydroxybenzoic acids, nine hydroxycinnamic acids, and three flavanols were identified and quantified, most of them for the first time. Hydroxybenzoic acids were the major phenolic compounds in all chestnut cultivars/accessions, followed by flavanols, dicarboxylic acid derivatives, and hydroxycinnamic acids. Of all the compounds studied, the (epi)catechin dimer was the most abundant in chestnut. The assumption that cultivars from commercial hybrids have a better and different metabolic profile than “native” accessions was refuted.

## 1. Introduction

There are 12 chestnut species worldwide, four of which are economically important: *Castanea sativa* Mill. (sweet chestnut), *Castanea crenata* Siebold & Zucc. (Japanese chestnut), *Castanea mollissima* Blume (Chinese chestnut), and *Castanea dentata* (Marsh.) Borkh. (American chestnut). Overall, *C. sativa* is the largest fruit-bearing species and most widely consumed chestnut species [1]. In commercial orchards today, interspecific hybrids of *C. sativa* and *C. crenata* are mostly grown. The trees are either grown from cuttings or grafted onto rootstocks [2], but the majority of all chestnuts are still harvested from “native” chestnut groves, which are found in temperate zones, mainly in southern Europe and Asia, and have been maintained for decades [3]. These fruits of wild accessions are generally smaller and probably produce lower quality chestnuts with different metabolic profiles [2].

Aside from starch, fat, protein, fiber, minerals, and vitamins, chestnut kernels are believed to have numerous health-promoting properties. These benefits are thought to be due to the numerous phytonutrients, of which phenolic compounds are considered the most important group [4]. Similar to walnuts, chestnuts have very diverse phenolic content, but unlike other nuts, there is very little research on the phenolic content of chestnuts. Therefore, only limited and outdated information is available on the phenolic profile and phenolic content of chestnuts, both for the commercial interspecific hybrids and for the “native” *C. sativa*. The main polyphenols reported for chestnuts are flavanols and phenolic acids [5], although it should be noted that most studies are outdated and do not use modern precision instruments such as mass spectrometers. 

Thus, the objective of the present study was to determine the phenolic profile and quantify the phenolic compounds in the raw chestnuts of the three most commonly grown hybrids (‘Bouche de Betizac’, ‘Marsol’, and ‘Maraval’) and three “native” accessions of *C. sativa*. This information will provide new insight into the phenolic profile of chestnut, as the available information is very sparse and outdated, and very little is known about the phenolic profile of chestnut (only one study on the phenolic compounds using a mass spectrometer in chestnuts was found looking at the literature [5]; however, only one cultivar was used, and the focus of this study was more on the effect of different processing methods on the phenolic profile of chestnut products and not the raw chestnut), let alone the differences in phenolic profiles of the “native” accessions compared to the commonly used commercial hybrids.

## 2. Results and Discussion

### 2.1. Identification of Individual Dicarboxylic Acid Derivatives and Phenolic Compounds

**Figure 1:**
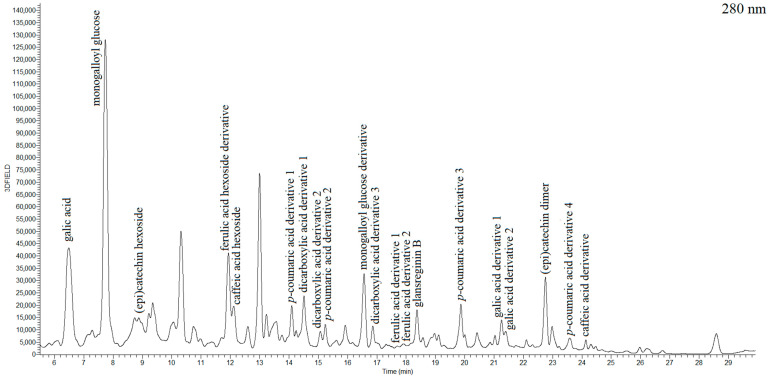
Chromatogram of a chestnut recorded at 280 nm.

Based on the mass spectra and literature, a total of 20 compounds were identified in our study in these chestnut samples, including 4 dicarboxylic acid derivatives, 5 hydroxybenzoic acids, 9 hydroxycinnamic acids, and 3 flavanols. Dicarboxylic acid derivatives and phenolic compounds were identified based on the mass-to-charge (*m*/*z*) ratio of the molecular ions and their characteristic fragment ions. Compounds for which no standards were available were fragmented based on their pseudomolecular ion [M-H]^−^ and specific fragmentation patterns (MS^2^, MS^3^, MS^4^). All data for the identified compounds are summarized in Table 1, including their *m*/*z*, relative abundance of fragment ions, MS/MS fragmentation, and standards used. In Figure 1, the chromatogram of the identified compounds can be seen. In Supplementary Materials (Figures S1–S7), total ion current chromatogram, base peak chromatogram and ion chromatograms with fragmentation spectra of gallic acid, monogalloyl glucose, caffeic acid hexoside, *p*-coumaric acid derivative and glansreginin B can be seen.

The dicarboxylic acid derivatives identified in chestnut were characterized by their typical fragmentation pattern, which produced the fragments *m*/*z* 241 and 197, as previously reported in peeled walnut kernels by Medic et al. [6]. In addition, glansreginin B was also identified by its typical fragmentation pattern of *m*/*z* 403, 343, and 241, which is consistent with that previously reported for walnut kernels [6,7,8]. The structure of glansreginin B has been assigned to the sucrose ester of glansreginic acid, which is a dicarboxylic acid derivative thought to be formed from tetraterpenoids in higher plants (i.e., violaxanthin) similar to abscisic acid [9]. Dicarboxylic acid derivatives have been previously detected in peeled walnut kernels [6] but were detected for the first time in chestnuts or any other nuts than walnuts.

Monogalloyl glucose and monogalloyl glucose derivatives were identified by their typical fragment loss of glucose (*m*/*z* 162) and typical fragment ions of *m*/*z* 331, 169, and 125, which is in agreement with Medic et al. [6] and Chen et al. [10], that have been reported in walnut kernels and in *Loropetalum chinense* (R.Br.) Oliv. To the best of our knowledge, none of these compounds have been previously reported for chestnuts. Gallic acid and two gallic acid derivatives were identified based on their typical fragmentation ions with *m*/*z* of 169 and 125 as reported by Singh et al. [11].

Two identified flavanols were identified as (epi)catechin derivatives following the specific fragmentation pattern of (−)epicatechin and (+)catechin, *m*/*z* 245, 205, and 179, as previously reported for other (epi)catechin derivatives in different walnut tissues by Medic et al. [12]. Monomeric flavanols or catechins are characterized by having a C_6_-C_3_-C_6_ skeleton with a hydroxyl group in position three of the C ring. Catechins rarely occur in nature in their glycosylated form, but their polymerized forms and derivatives are frequently found in plant foods [13], as also shown in our study.

The nine hydroxycinnamic acids were mainly ferulic acid, caffeic acid, and *p*-coumaric acid derivatives. All followed the characteristic fragmentation patterns (ferulic acid, MS^n^ ion *m*/*z* 193 and MS^n+1^ ion *m*/*z* 149; caffeic acid, MS^n^ ion *m*/*z* 179 and MS^n+1^ ion *m*/*z* 135; *p*-coumaric acid, MS^n^ ion *m*/*z* 163 and MS^n+1^ ion *m*/*z* 119), as previously reported by Vieira et al. [14] and Šuković et al. [15] and confirmed by fragmentation of the standards (ferulic acid, caffeic acid, and *p*-coumaric acid).

### 2.2. Content of Dicarboxylic Acid Derivatives and Phenolic Compounds in Chestnuts

Hydroxybenzoic acids were the major phenolic compounds in all chestnut cultivars/accessions. This group accounted for between 53.9% (‘A2’) and 85.9% (‘A3’) of the TAPC, with contents ranging from 86.44 mg/kg FW (fresh weight) to 185.54 mg/kg FW. Interestingly, the highest and lowest levels of hydroxybenzoic acids were reported for *C. sativa* accessions, suggesting that the genetic diversity, as well as the phenolic profile, were highly variable compared to the commercial cultivars studied, whose levels of hydroxybenzoic acids ranged from 79.1% (‘Maraval’) to 83.9% (‘Bouche de Betizac’). The content of hydroxybenzoic acids in our study was higher than in the previous study on raw chestnuts by Mustafa et al. [5]; this could be influenced by different climatic conditions or agrotechnical management or the lack of previous identification and quantification of the compounds in the chestnut itself. Of the hydroxybenzoic acids, monogalloyl glucose was the most abundant hydroxybenzoic acid, accounting for up to 83% of the total hydroxybenzoic acid content. Monogalloyl glucose has previously been described as the most abundant phenolic compound in the peeled walnut kernels [6], as is now the case in the chestnut in our study. It has previously not been identified or quantified in chestnuts; therefore, any comparisons are not plausible. Overall, hydroxybenzoic acids are related to salicylic acid and salicin, one of the first isolated compounds to have pharmacological activity. These compounds are thought to activate hydroxycarboxylic acid receptors, which may lead to a reduction in lipolysis in adipocytes and thus an improvement in blood lipid profiles. Several of the other compounds may activate the Nrf2 pathway, which increases the expression of antioxidant enzymes, thereby reducing oxidative stress and associated problems such as endothelial dysfunction, which leads to hypertension, and general inflammation [16].

The second most represented group was flavanols, both in our study and in the study of Mustafa, et al. [5], although the content of flavanols in our study was slightly lower, ranging from 9.64 mg/kg FW (‘A1’) to 78.97 mg/kg FW (‘A2’). Flavanols consumption is thought to have cardioprotective effects by influencing antithrombotic mechanisms, endothelial cell function and blood pressure [17,18]. Of the flavanols, the (epi)catechin dimer was the most abundant compared to the (+)catechin previously reported by Mustafa, et al. [5]. As mentioned earlier, catechins rarely occur in nature in their glycosylated form, but their polymerized forms and derivatives are commonly found [13], making (epi)catechin dimer, the most abundant flavanol, more logical than (+)catechin or (−)epicatechin.

The third most abundant group was dicarboxylic acid derivatives, which were the most abundant group of compounds identified in the peeled walnut kernels [6]. Of the four dicarboxylic acid derivatives, three were identified and quantified for the first time in chestnuts or any other nuts or organisms. The only previously known compound was glansreginin B, which was the second most abundant dicarboxylic acid derivative detected in peeled walnut kernels. Interestingly, glansreginin A, the most abundant dicarboxylic acid derivative in peeled walnut kernels, was not found even in trace amounts in chestnuts. The highest level of dicarboxylic acid derivatives was reported for ‘A1’ (22.29 mg/kg FW). No comparisons could be made with other chestnuts, since this was the first time that dicarboxylic acid derivatives were detected in chestnuts; however, in comparison with walnuts, where dicarboxylic acid derivatives have been detected previously [6], the content in peeled walnut kernels is up to 100-times higher than in chestnuts, while the content of glansreginin B in chestnuts is up to 10-times lower compared to peeled walnut kernels.

Finally, the TAPC varied from 121.17 mg/kg FW (‘A1’) to 234.41 mg/kg FW (‘Maraval’), which is in agreement with Mustafa et al. [5] (165.35 mg/kg DW). Interestingly, the TAPC in chestnuts is comparable to that in peeled walnut kernels, which are believed to have the highest TAPC among tree nuts [19]. However, since chestnuts are consumed peeled and walnuts are consumed with the pellicle (unpeeled), which accounts for more than 90% of the phenolic compounds per walnut, the content per nut consumed is much higher for walnuts compared to chestnuts. However, the phenolic profile of chestnut compared to peeled walnut kernel is more diverse, as peeled walnut kernels contain mainly dicarboxylic acid derivatives [6], while in chestnuts, dicarboxylic acid derivatives account for less than 18% of TAPC, following by hydroxybenzoic acids and flavanols. Overall, there were no clear results to suggest that all the interspecific hybrid cultivars studied had higher or lower TAPC than the “native” *C. sativa* accessions studied. Therefore, the assumption of Massantini et al. [2] that cultivars of commercial hybrids have a better and different metabolic profile than “native” accessions could be refuted. The variability of “native” accessions in terms of metabolic profile is indeed higher than cultivars of commercial hybrids, but their metabolic profile is comparable in terms of both metabolite profile and content. Although no nutritional functions are attributed to phenolic compounds, they are still very important for human health due to their antioxidant, anti-inflammatory, antimutagenic, and antiatherogenic properties [20]. Therefore, the consumption of chestnuts in general is recommended. All individual contents, as well as the total phenolic contents, are shown in Table 2. 

## 3. Materials and Methods

### 3.1. Samples

Chestnuts of the three commercial cultivars (‘Bouche de Betizac’, ‘Marsol’, and ‘Maraval’) were collected from an Experimental Field for Nut Crops in Maribor (Slovenia; 46°34′01″ N; 15°37′51″ E; 280 m a.s.l.), and samples of “native” accessions (labelled ‘A1’, ‘A2’, and ‘A3’) were collected 5 km away near Bresternica (Slovenia; 46°34′20″ N 15°35′35″ E; 320 m a.s.l.). They were collected as close as possible to the Experimental Field for Nut Crops in Maribor to have the same soil and climate. The chestnuts were collected at their technological stage of maturity, after the burrs had split and the chestnuts had fallen to the ground with or without burrs. After collection, the samples were taken to the laboratory of the Department of Agronomy of the Biotechnical Faculty of the University of Ljubljana (Slovenia), where they were further analyzed.

### 3.2. Extraction of Dicarboxylic Acid Derivatives and Phenolic Compounds 

The phenolic compounds and dicarboxylic acid derivatives were extracted according to the protocol described by Medic et al. [6]. Twenty chestnuts per cultivar were used, with five chestnuts per replicate. The chestnuts were weighed; then, the seed coat was removed, and the pellicles were peeled. The peeled raw chestnuts were then ground using a mechanical mill (A10 basic; IKA Works GmbH & Co. KG, Staufen, Germany). Briefly, 1 g of the sample was then extracted with 80% methanol in bi-distilled water. The extraction ratio was 1:2.5 (*w*/*v*). The samples were then vortexed (TOP-MIX 94500 vortex mixer; Heidolph, Schwabach, Germany), sonicated in iced water for 60 min (Sonis 4 ultrasonic bath; Iskra pio, Sentjernej, Slovenia), and centrifuged at 10,000× *g* for 10 min at 4 °C. Samples were then filtered with a 0.2 µm polyamide filter (Chromafil AO −20/25; Macherey-Nagel, Düren, Germany), transferred to vials, and stored at −20 °C for further analysis.

### 3.3. HPLC–MS Analysis of Dicarboxylic Acid Derivatives and Phenolic Compounds

For the identification and quantification of phenolic compounds and dicarboxylic acid derivatives, we used a LTQ XL tandem mass spectrometer with heated electrospray ionization operated in negative ion mode and coupled to a Vanquish UHPLC system (Thermo Scientific, Waltham, MA, USA) with a diode array detector at 280 nm. All parameters were used as described by Medic et al. [6]. The quantification of the different compounds is given using the different standards in Table 1. The compounds were quantified using the standards; where standards were not available, a similar standard was used. Total analyzed phenolics content, referred to as TAPC in the text, represents the sum of all phenols identified and is expressed in mg/kg fresh weight.

### 3.4. Chemicals

The following standards were used for the identification and quantification of dicarboxylic acid derivatives and phenolic compounds: (+)catechin (Roth, Karlsruhe, Germany); caffeic acid, gallic acid, ellagic acid, *p*-coumaric acid, ferulic acid (Sigma-Aldrich Chemie GmbH, Steinheim, Germany).

Acetonitrile for the mobile phases was HPLC-MS grade (Fluka Chemie GmbH, Buchs, Switzerland). Methanol and formic acid were HPLC grade (Sigma-Aldrich Chemie GmbH, Steinheim, Germany). The bi-distilled water was purified using a water purification system (Milli-Q, Millipore, Bedford, MA, USA).

### 3.5. Statistical Analysis

Data were collected using Microsoft Excel 2016 and statistically analyzed using R commander (package Rcmdr) version 2.7.1. (Team, R.D.C.; 2008, Stanford, CA, USA). Five chestnut samples per accession/cultivar were examined, and four replicates of each methodology were performed. One-way analysis of variance (ANOVA) with Tukey tests was performed to determine significant differences between data. All data are presented as means ± standard errors (SE). Statistical means were calculated at the 95% confidence level to determine the significance of differences (*p* < 0.05).

## 4. Conclusions

A total of 20 compounds were identified and quantified in these chestnut samples, including 4 dicarboxylic acid derivatives and 16 phenolic compounds, most of them for the first time in chestnuts or other nuts. This is the first report of dicarboxylic acid derivatives in chestnuts. As far as we know, this is the most comprehensive study describing the content of various phenols and dicarboxylic acid derivatives in chestnut. Hydroxybenzoic acids were the most important phenolic compounds in all chestnut cultivars/accessions, followed by flavanols, dicarboxylic acid derivatives, and hydroxycinnamic acids. Of all the compounds studied, the (epi)catechin dimer was the most abundant in chestnut. The TAPC in chestnuts is comparable to that in peeled walnut kernels, which are thought to have the highest TAPC among tree nuts. There were no clear results to suggest that all interspecific hybrid cultivars studied had higher or lower TAPC than the “native” *C. sativa* accessions studied. The assumption that cultivars of commercial hybrids have a better and different metabolic profile than “native” accessions was refuted. The variability of “native” accessions in terms of metabolic profile is indeed higher than cultivars of commercial hybrids, but their metabolic profile is comparable in terms of both metabolite profile and content.

## Figures and Tables

**Table 1:** Tentative identification of the 20 phenolic compounds and dicarboxylic acid derivatives, as well as the standards that they are expressed relative to.

Compound	Rt (min)	[M-H]^−^ (*m*/*z*)	MS^2^ (*m*/*z*)	MS^3^ (*m*/*z*)	MS^4^ (*m*/*z*)	Expressed As
Gallic acid	6.48	169	125(100)	125(100), 81(38), 97(20)		Gallic acid
Monogalloyl glucose	7.73	331	169(100), 125(3)	125(100)		Gallic acid
(epi)Catechin hexoside	8.93	451	289(100)	245(100), 205(35), 179(13), 125(8)		(+)Catechin
Ferulic acid hexoside derivative	11.92	401	355(100), 193(21)			Ferulic acid
Caffeic acid hexoside	12.57	341	179(100), 135(5)	135(100)		Caffeic acid
*p*-Coumaric acid derivative 1	14.11	387	207(100), 163(47), 119(3)	163(100), 119(5)		*p*-Coumaric acid
Dicarboxylic acid derivative 1	14.46	565	241(100), 197(22)	197(100)		Ellagic acid
Dicarboxylic acid derivative 2	15.43	583	421(100), 515(43), 547(43), 241(31), 197(8)			Ellagic acid
*p*-Coumaric acid derivative 2	15.63	433	387	207(100), 163(47), 119(3)		*p*-Coumaric acid
Monogalloyl glucose derivative	16.56	375	357(100), 331(47), 307(30), 169(5)			Gallic acid
Dicarboxylic acid derivative 3	16.87	403	241(100), 197(14)			Ellagic acid
Ferulic acid derivative 1	17.71	308	193(100), 132(24), 264(19), 246(17)	134(100), 149(56), 178(32)		Ferulic acid
Ferulic acid derivative 2	18.13	322	304(100), **193**(23), 128(13)	134(100), 149(56), 178(32)		Ferulic acid
Glansreginin B	18.37	565	403(100), 241(30), 343(28), 341(21), 197(7)			Ellagic acid
*p*-Coumaric acid derivative 3	19.87	431	389(100), 163(91), 371(56), 125(22)			*p*-Coumaric acid
Gallic acid derivative 1	21.24	643	625(100), 607(86), 589(16)	381(100), 406(76)	261(100), 169(18), 245(15), 181(11), 125(4)	Gallic acid
Gallic acid derivative 2	21.40	481	313(100), 167(21), 271(20)	169(100), 125(83)		Gallic acid
(epi)Catechin dimer	22.75	579	245(100), 495(65), 289(38), 271(21), 203(13)			(+)Catechin
*p*-Coumaric acid derivative 4	23.58	279	163(100)	119(100)		*p*-Coumaric acid
Caffeic acid derivative	24.31	544	363(100), 364(90), 500(45), 345(30), 346(27)	345(100), 335(97), 179(14), 135(2)		Caffeic acid

First or bold number, fragments that were further fragmented; Rt, retention time; [M-H]^−^, pseudomolecular ion identified in negative ion mode; (), relative abundance of fragment ions; MS^2^, fragment ions obtained from pseudomolecular ion in negative ion mode; MS^3^, fragment ions obtained from the most abundant or bolded pseudomolecular ion of MS^2^ fragmentation; MS^4^, fragment ions obtained from the most abundant or bolded pseudomolecular ion of MS^3^ fragmentation.

**Table 2:** Dicarboxylic acid derivatives and phenolic compounds found in chestnuts (three commercial interspecific hybrids (*C. sativa* x *C. crenata*) and three accessions of *C. sativa*).

Compound	Quantification According to Cultivar (mg/kg Fresh Weight)					
	‘Bouche de Betizac’	‘Marsol’	‘Maraval’	‘A1’	‘A2’	‘A3’
*Dicarboxylic acid derivatives*						
Glansreginin B	6.97 ± 0.78 ab	10.04 ± 0.95 b	6.05 ± 0.75 a	16.23 ± 1.38 c	11.71 ± 2.91 b	5.48 ± 1.03 a
Dicarboxylic acid derivative 1	1.03 ± 0.26 ab	0.44 ± 0.18 a	0.29 ± 0.04 a	0.47 ± 0.08 ab	2.02 ± 0.46 b	0.06 ± 0.00 a
Dicarboxylic acid derivative 2	1.00 ± 0.49 b	1.00 ± 0.14 b	1.07 ± 0.02 b	1.44 ± 0.40 b	1.08 ± 0.68 b	0.18 ± 0.03 a
Dicarboxylic acid derivative 3	9.05 ± 1.18 d	2.20 ± 0.25 ab	1.48 ± 0.21 a	4.15 ± 0.70 c	4.79 ± 1.34 c	3.15 ± 0.37 bc
*Hydroxycinnamic acids*						
Ferulic acid hexoside derivative	0.33 ± 0.04 c	0.01 ± 0.01 a	0.14 ± 0.01 b	0.03 ± 0.02 a	0.07 ± 0.03 ab	0.31 ± 0.02 c
Ferulic acid derivative 1	0.17 ± 0.07 a	0.06 ± 0.00 a	0.11 ± 0.01 a	0.16 ± 0.02 a	0.35 ± 0.12 a	0.26 ± 0.09 a
Ferulic acid derivative 2	0.06 ± 0.01 a	0.02 ± 0.00 a	0.09 ± 0.02 a	0.12 ± 0.04 a	0.09 ± 0.04 a	0.10 ± 0.01 a
Caffeic acid hexoside	0.79 ± 0.42 b	0.00 ± 0.00 a	0.36 ± 0.05 ab	0.05 ± 0.02 a	0.04 ± 0.01 a	0.41 ± 0.2 ab
Caffeic acid derivative	0.06 ± 0.01 a	0.12 ± 0.01 a	0.08 ± 0.01 a	0.66 ± 0.08 b	2.19 ± 0.05 c	0.01 ± 0.00 a
*p*-Coumaric acid derivative 1	0.17 ± 0.06 b	0.05 ± 0.02 a	0.10 ± 0.04 a	0.17 ± 0.05 b	0.11 ± 0.02 b	0.01 ± 0.01 a
*p*-Coumaric acid derivative 2	0.10 ± 0.09 ab	0.11 ± 0.01 ab	0.03 ± 0.01 a	0.10 ± 0.02 ab	0.26 ± 0.04 b	0.05 ± 0.02 a
*p*-Coumaric acid derivative 3	2.01 ± 0.48 b	0.49 ± 0.13 a	0.98 ± 0.13 a	1.43 ± 0.16 ab	0.97 ± 0.09 a	0.88 ± 0.26 a
*p*-Coumaric acid derivative 4	0.25 ± 0.03 c	0.05 ± 0.00 a	0.24 ± 0.01 b	0.09 ± 0.01 a	0.37 ± 0.01 c	0.09 ± 0.00 a
*Hydroxybenzoic acids*						
Gallic acid	18.87 ± 4.08 b	12.49 ± 2.63 a	27.98 ± 2.27 c	24.71 ± 3.27 bc	13.50 ± 1.21 a	10.02 ± 2.22 a
Gallic acid derivative 1	7.16 ± 0.37 b	2.85 ± 0.19 a	3.70 ± 0.31 a	10.92 ± 1.53 c	9.77 ± 0.48 bc	10.51 ± 4.85 c
Gallic acid derivative 2	4.99 ± 0.95 ab	0.97 ± 0.05 a	1.55 ± 0.16 a	3.10 ± 0.33 ab	2.81 ± 0.31 ab	7.95 ± 3.73 b
Monogalloyl glucose	121.05 ± 11.90 c	112.89 ± 4.99 bc	143.80 ± 6.79 d	34.65 ± 3.14 a	78.73 ± 5.33 b	108.33 ± 5.39 bc
Monogalloyl glucose derivative	19.49 ± 3.11 bc	6.15 ± 0.79 a	8.50 ± 1.03 ab	13.06 ± 3.28 ac	15.57 ± 1.59 ac	10.21 ± 1.09 ab
*Flavanols*						
(epi)Catechin hexoside	1.26 ± 0.06 d	0.80 ± 0.04 d	1.61 ± 0.05 c	0.25 ± 0.02 ab	0.11 ± 0.04 a	0.50 ± 0.14 bc
(epi)Catechin dimer	9.57 ± 2.51 a	13.87 ± 1.19 a	36.25 ± 0.95 b	9.39 ± 1.35 a	78.87 ± 2.07 c	12.66 ± 1.91 a
Total dicarboxylic acid derivatives	18.05 ± 0.91 b	13.68 ± 0.59 ab	8.89 ± 0.45 a	22.29 ± 1.05 c	19.60 ± 1.94 b	8.87 ± 0.91 a
Total hydroxycinnamic acids	3.95 ± 0.35 c	0.91 ± 0.15 a	2.12 ± 0.20 ab	2.81 ± 0.17 b	4.44 ± 0.21 d	2.13 ± 0.34 ab
Total hydroxybenzoic acids	171.57 ± 21.88 c	135.34 ± 9.63 b	185.54 ± 11.40 c	86.44 ± 13.01 a	120.38 ± 5.16 b	147.02 ± 8.72 bc
Total Flavanols	10.83 ± 2.29 a	14.67 ± 1.24 a	37.87 ± 0.93 b	9.64 ± 1.37 a	78.97 ± 2.03 c	13.17 ± 1.78 a
TAPC	204.40 ± 24.42 c	164.61 ± 11.00 b	234.41 ± 12.36 d	121.17 ± 14.29 a	223.40 ± 6.80 d	171.18 ± 10.22 b

Data are means ± standard error. TAPC, sum of all of the individual identified phenolics (summation). Means followed by different letters across the cultivars (within rows) are significantly different (*p* < 0.05).

## Data Availability

The remaining data presented in this study are available on request from the corresponding author. The remaining data are not publicly available due to privacy.

## Supplementary Materials

The following supporting information can be downloaded at: https://www.mdpi.com/article/10.3390/ijms241713086/s1.
