# Supplementary material for: Identification and Quantification of the Major Phenolic Constituents in Castanea sativa and Commercial Interspecific Hybrids (C. sativa x C. crenata) Chestnuts Using HPLC–MS/MS
Source: Int J Mol Sci. 2023 Aug 23;24(17):13086. doi: 10.3390/ijms241713086 (PMC10488303; doi:10.3390/ijms241713086)

Figure S1. Total ion current chromatogram

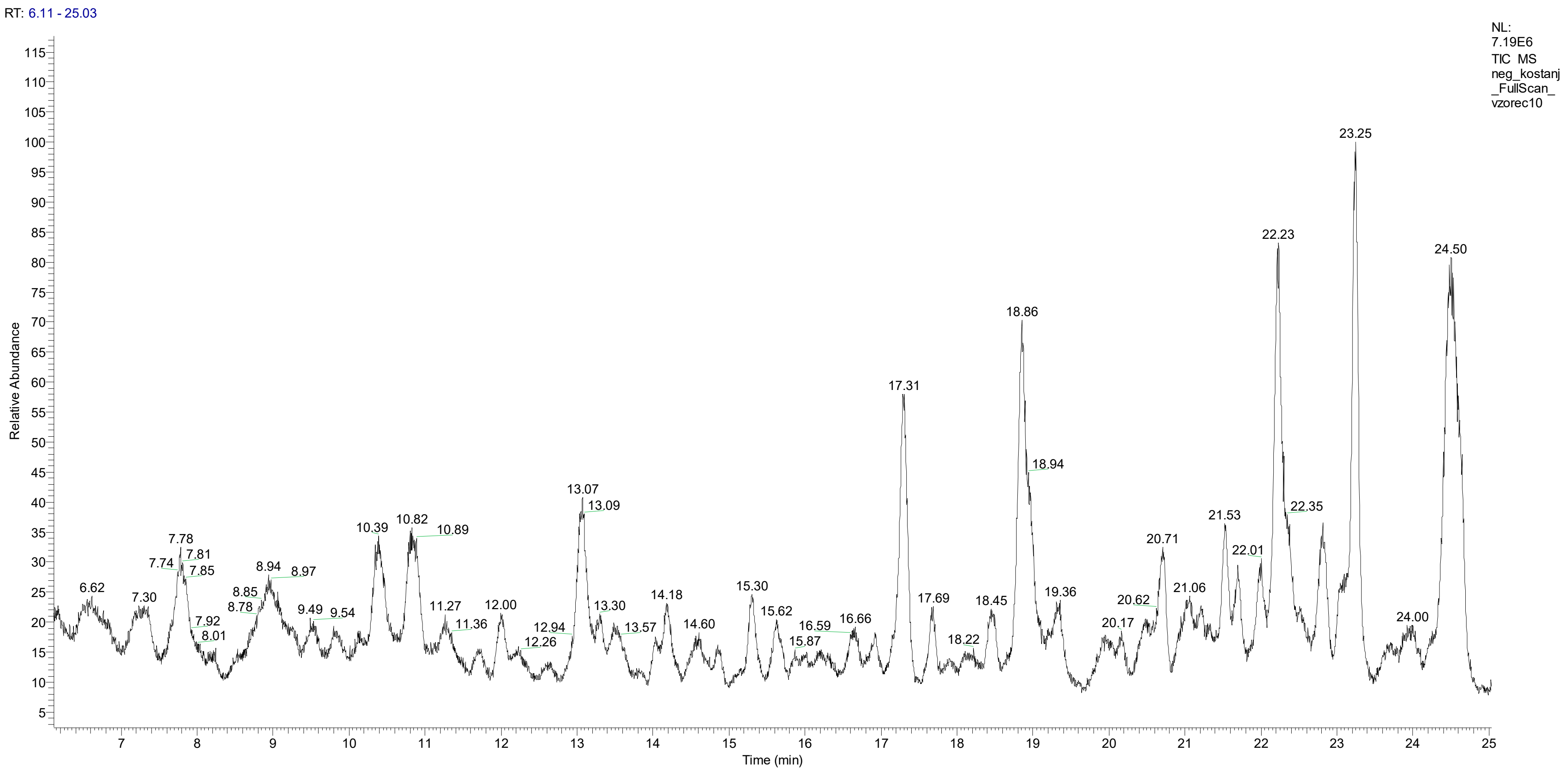

Figure S2. Base peak chromatogram

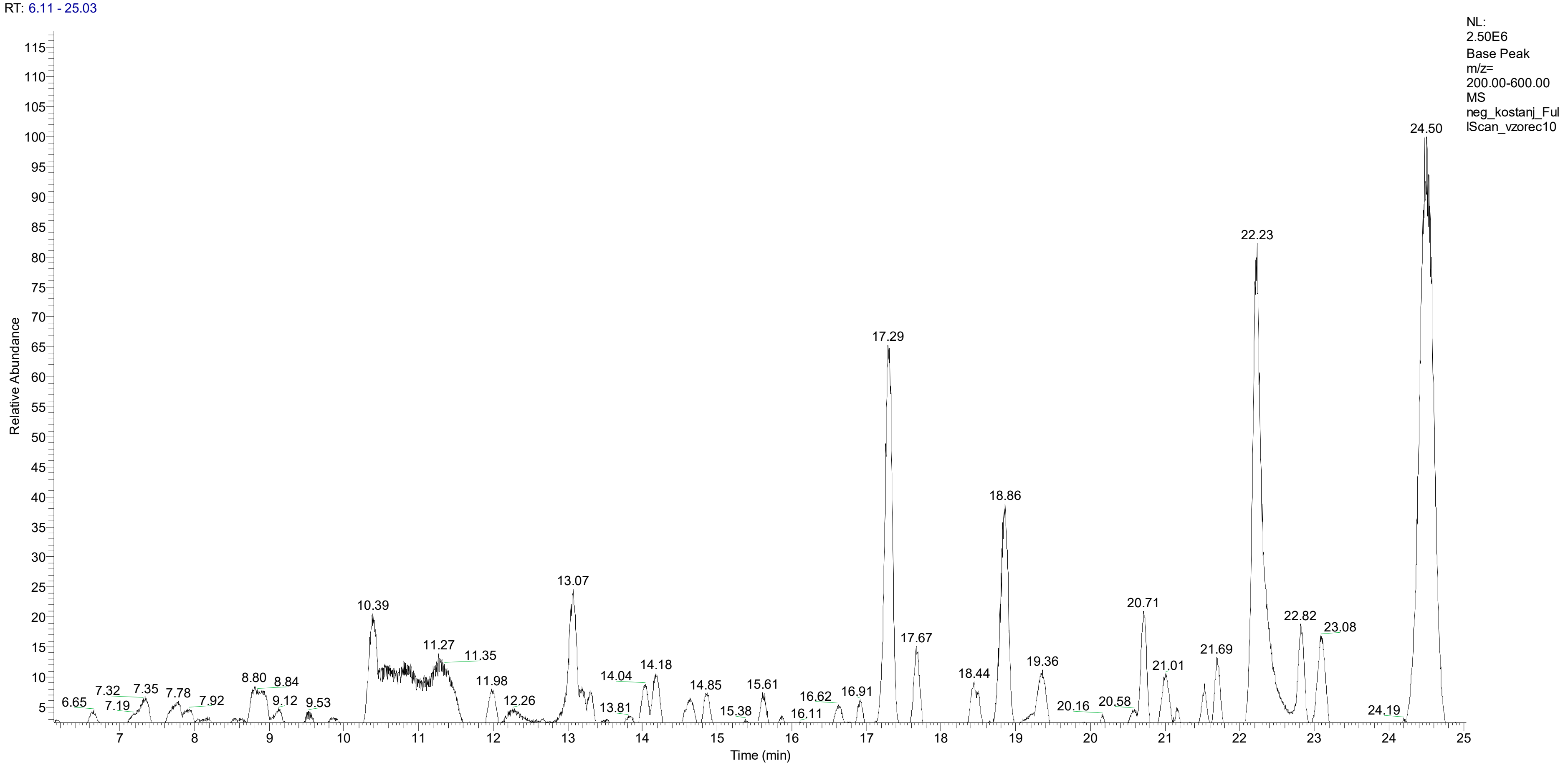

Figure S3a. Ion chromatogram of gallic acid

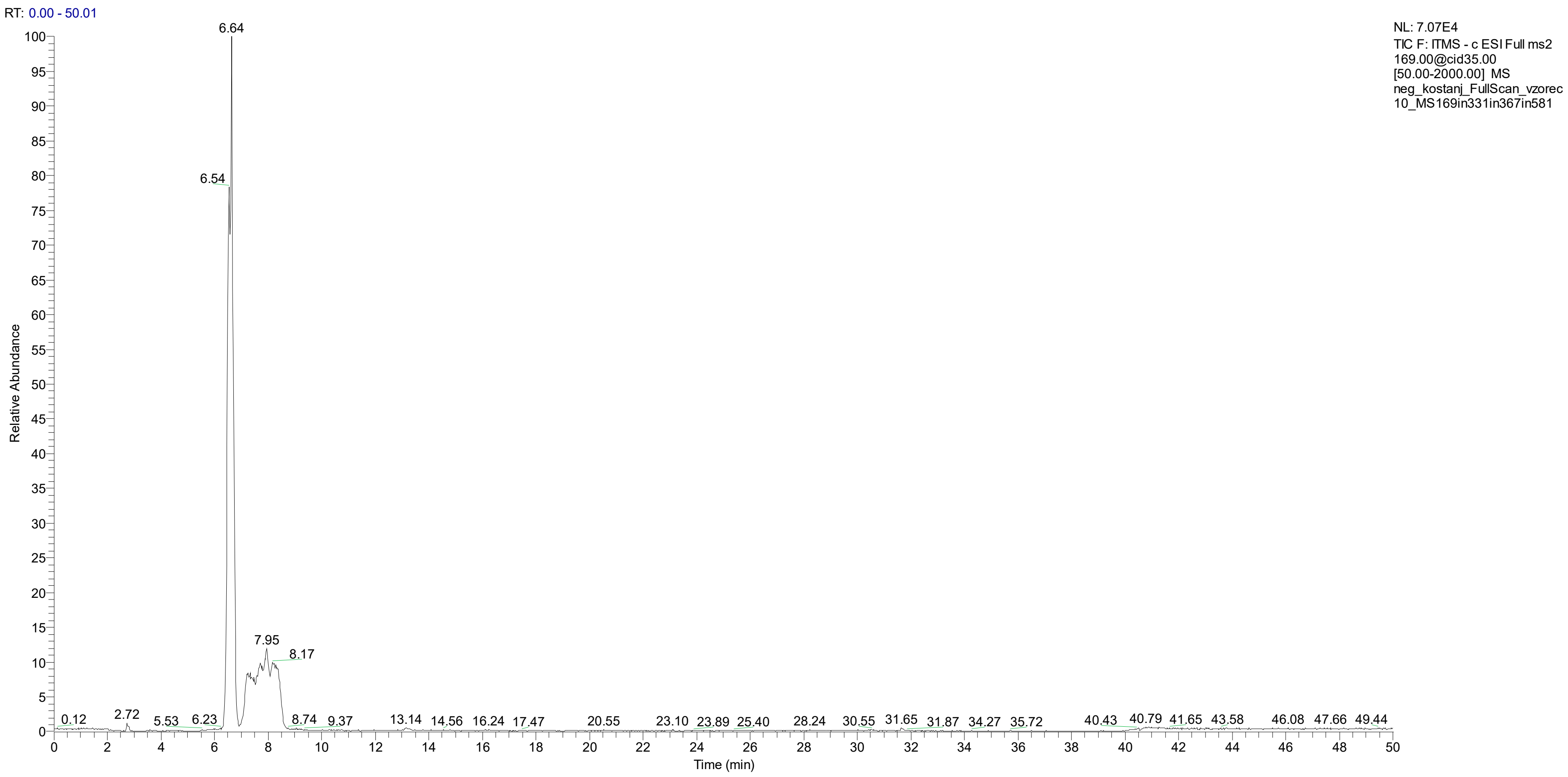

Figure S3b. Fragmentation spectra of gallic acid

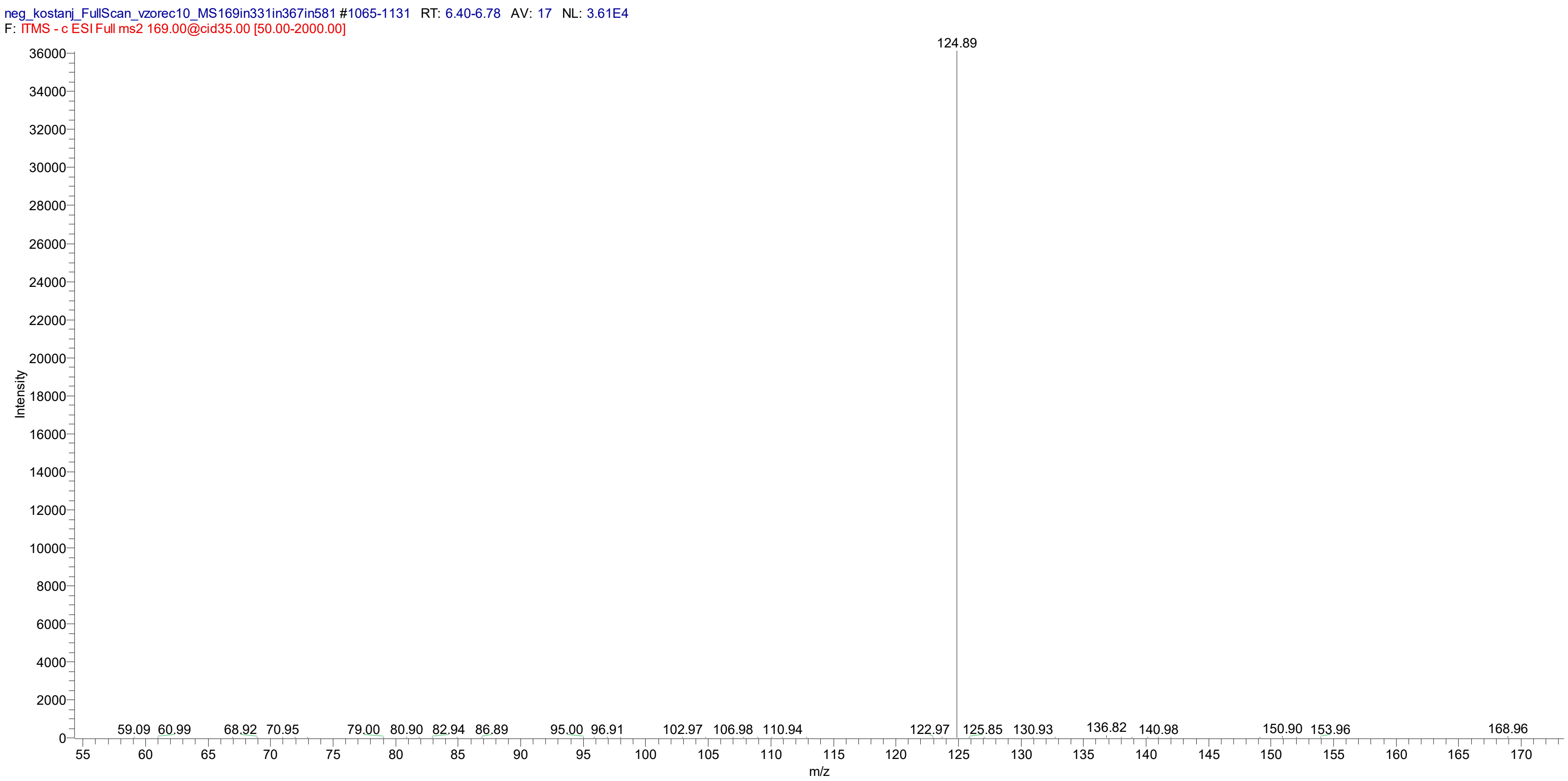

Figure S4a. Ion chromatogram of monogalloyl glucose

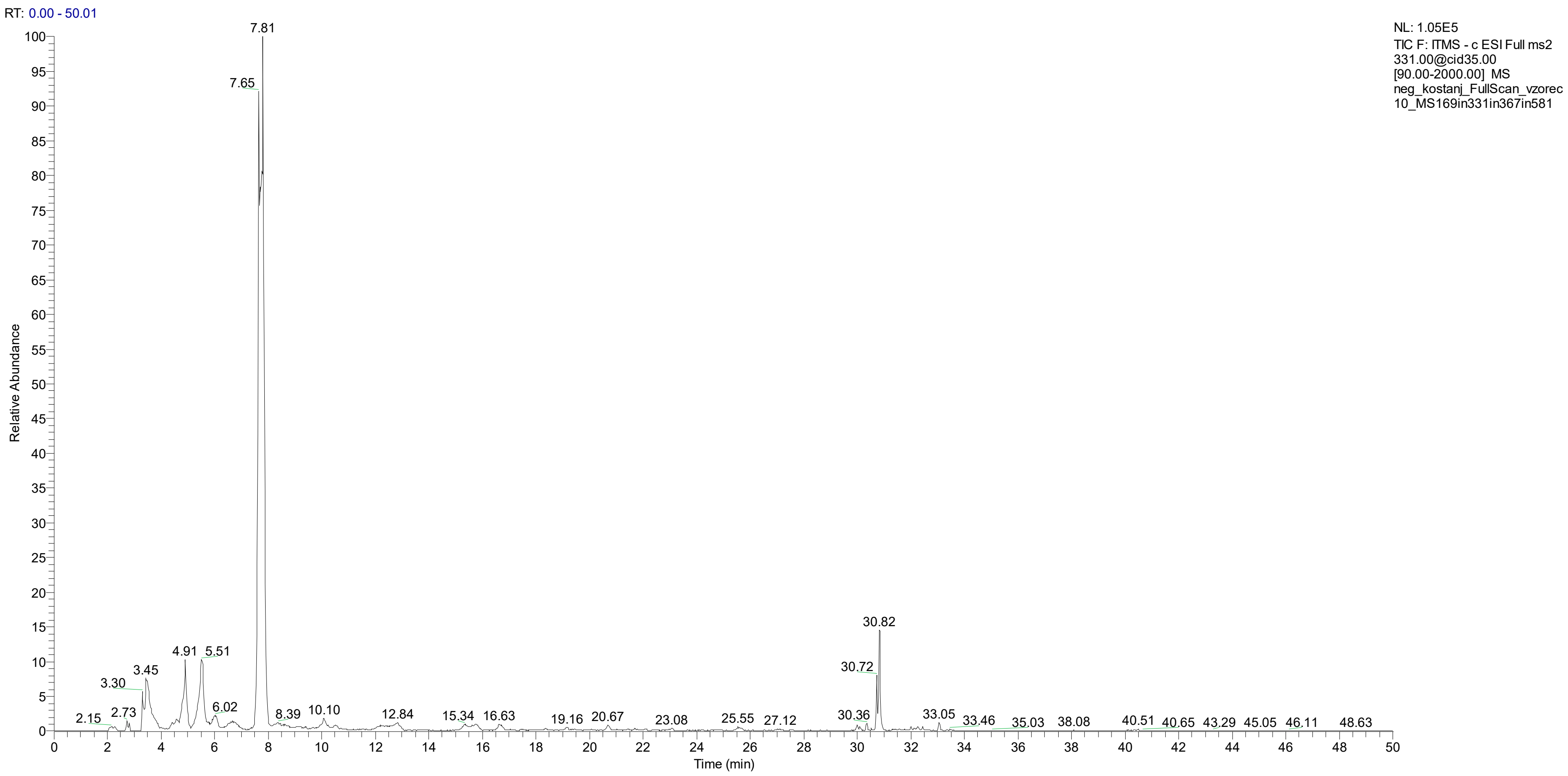

Figure S4b. Fragmentation spectra of monogalloyl glucose

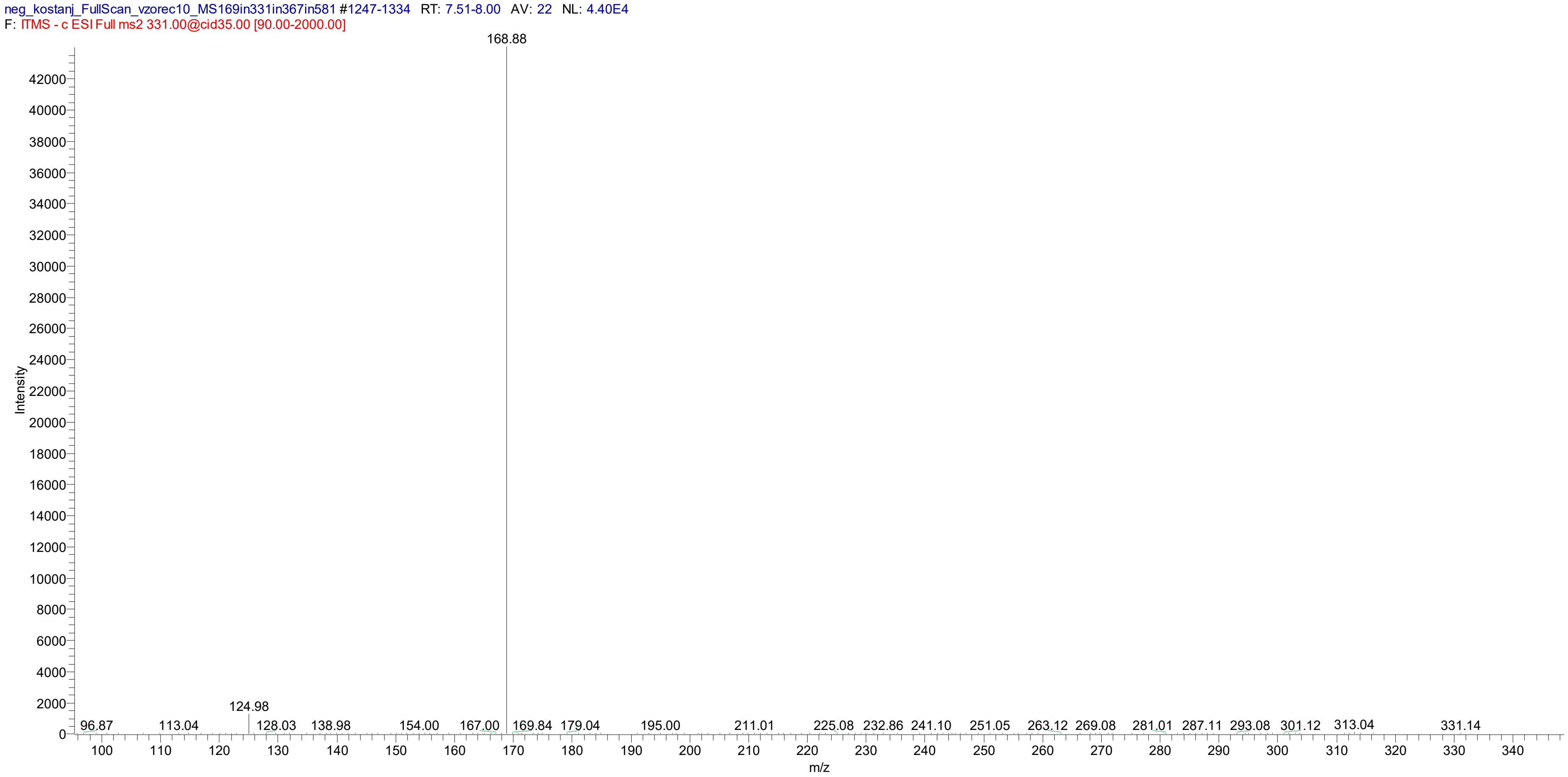

Figure S5a. Ion chromatogram of caffeic acid hexoside

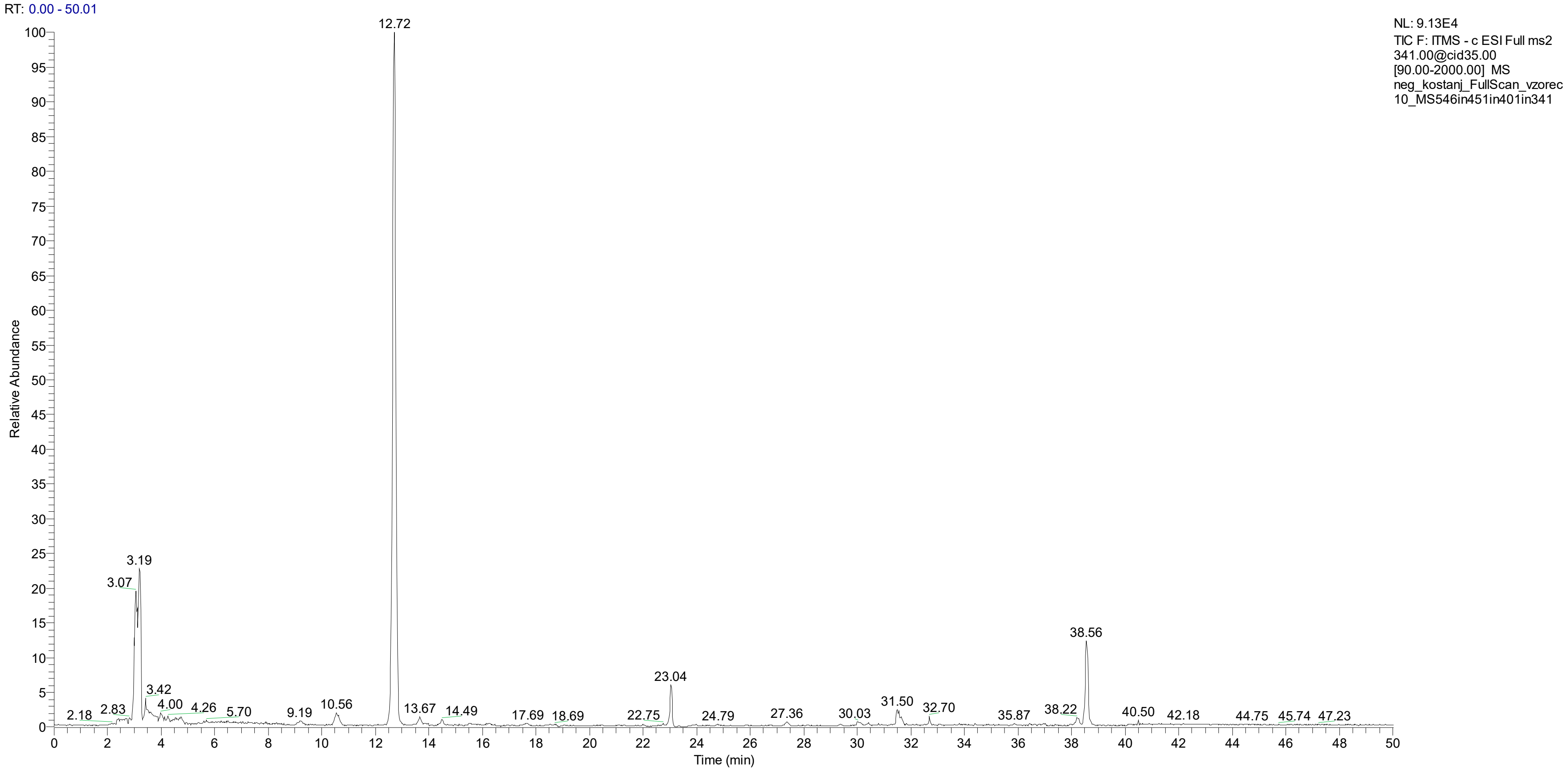

Figure S5b. Fragmentation spectra of caffeic acid hexoside

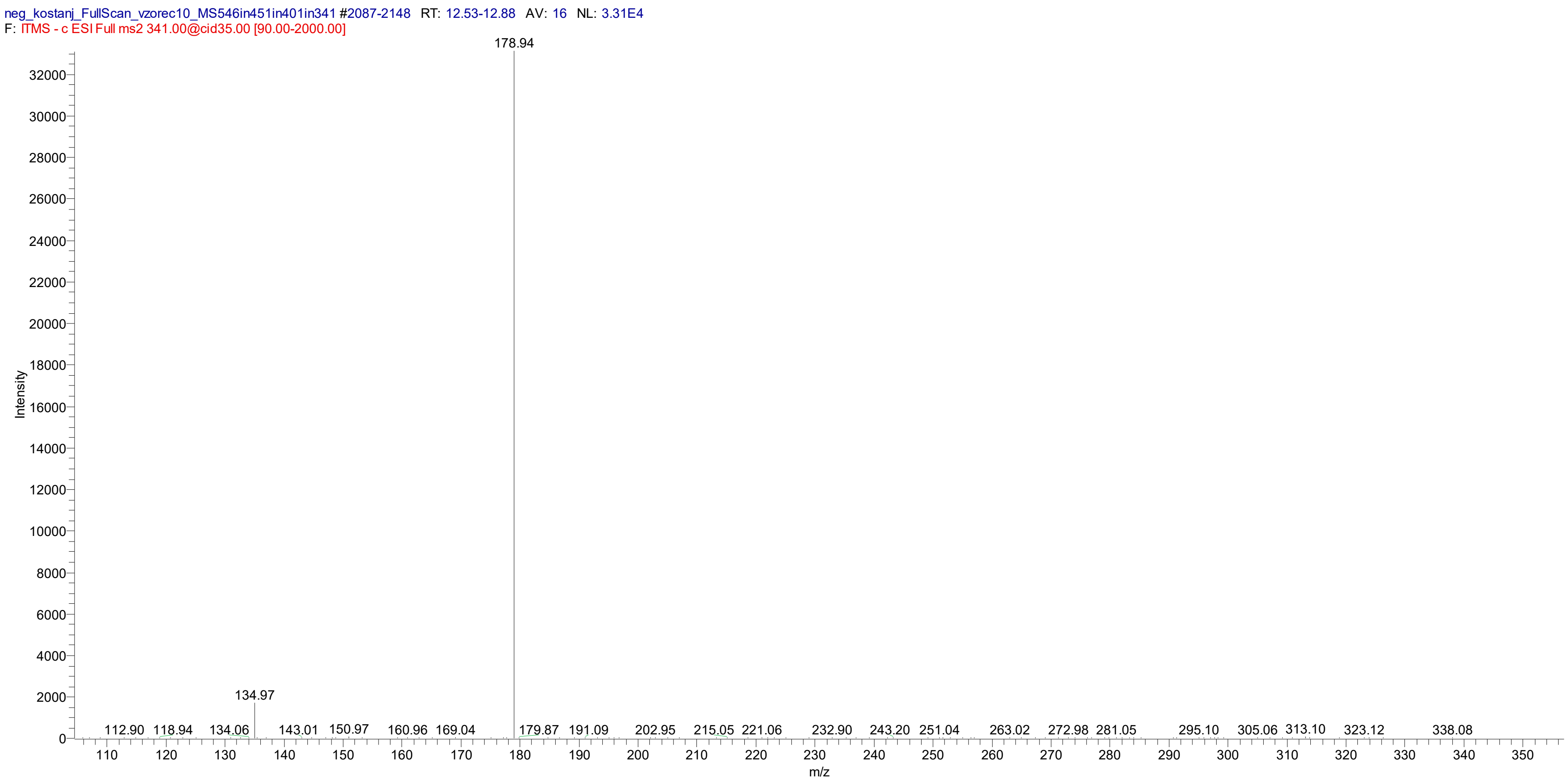

Figure S6a. Ion chromatogram of *p*-coumaric acid derivative

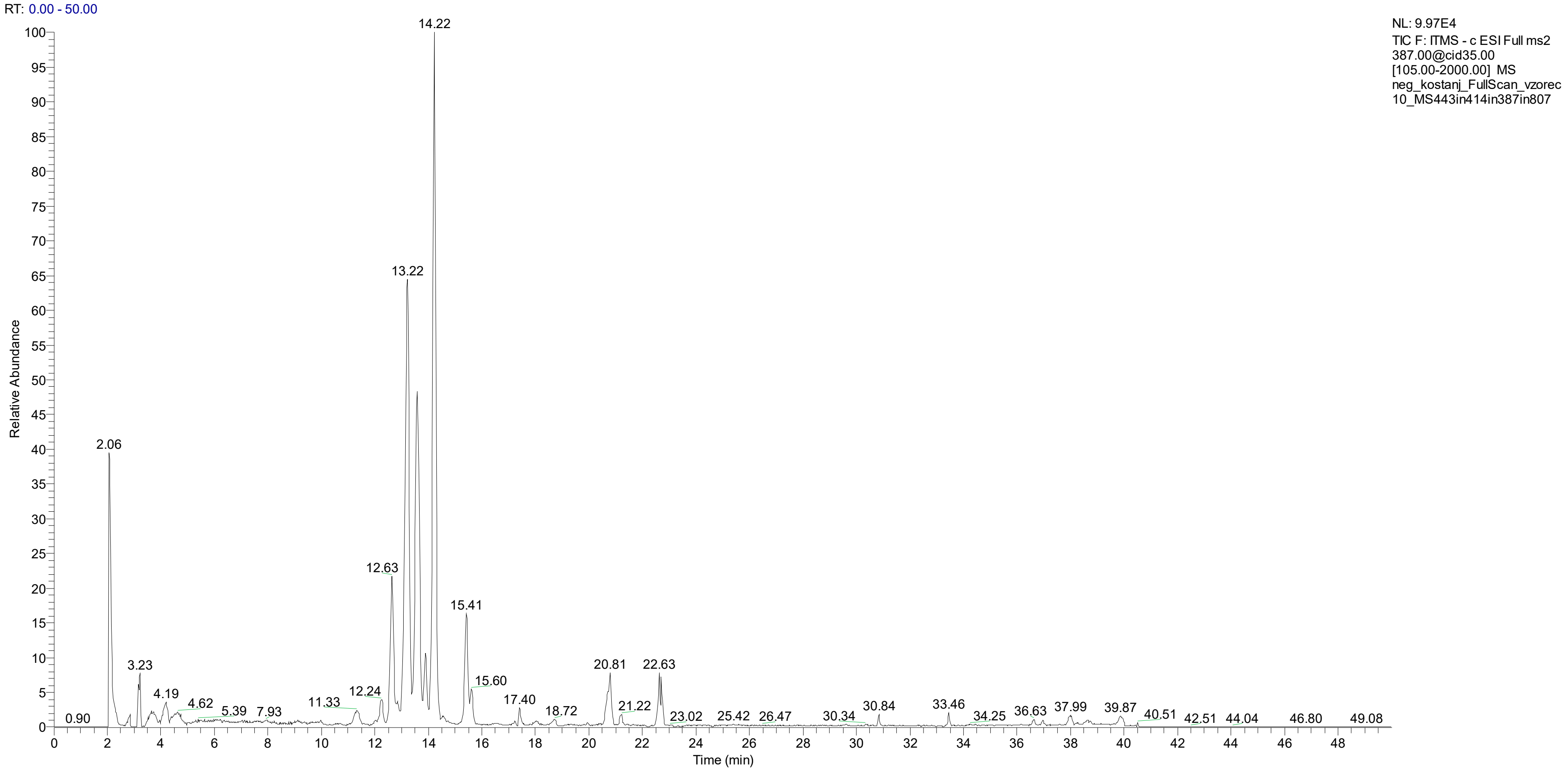

Figure S6b Fragmentation spectra of *p*-coumaric acid derivative

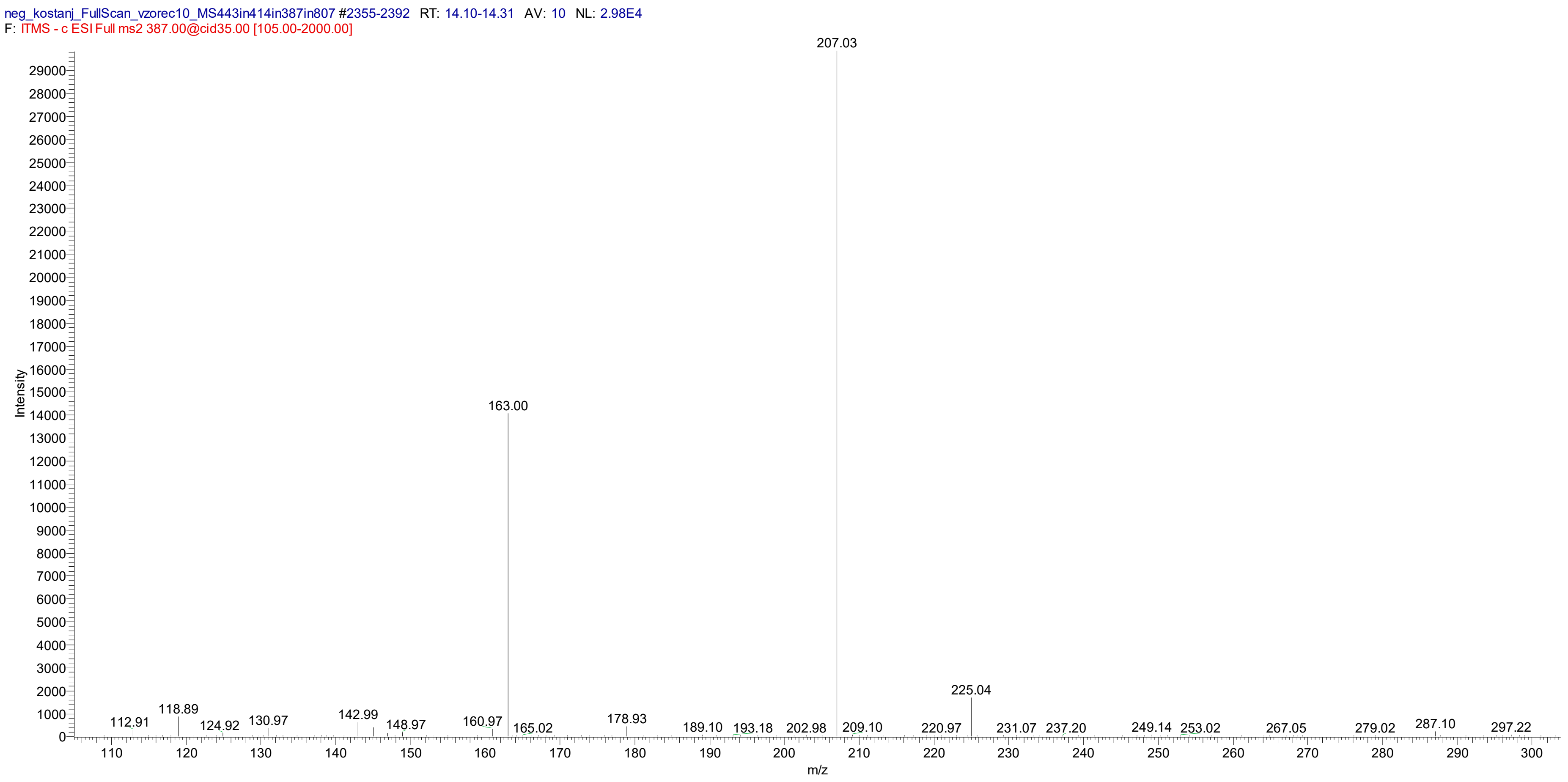

Figure S7a. Ion chromatogram of glansreginin B

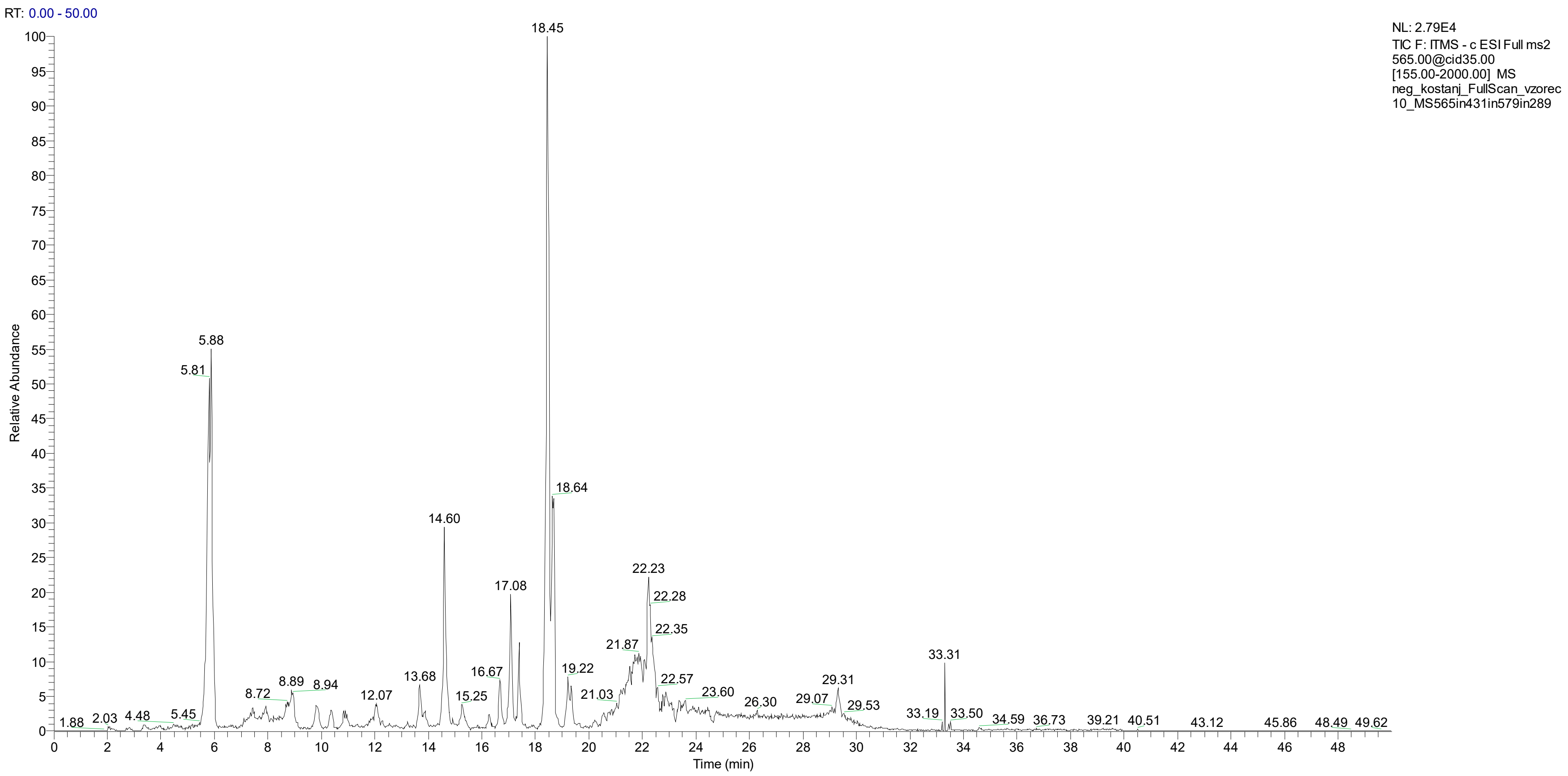

Figure S7b Fragmentation spectra of glansreginin B

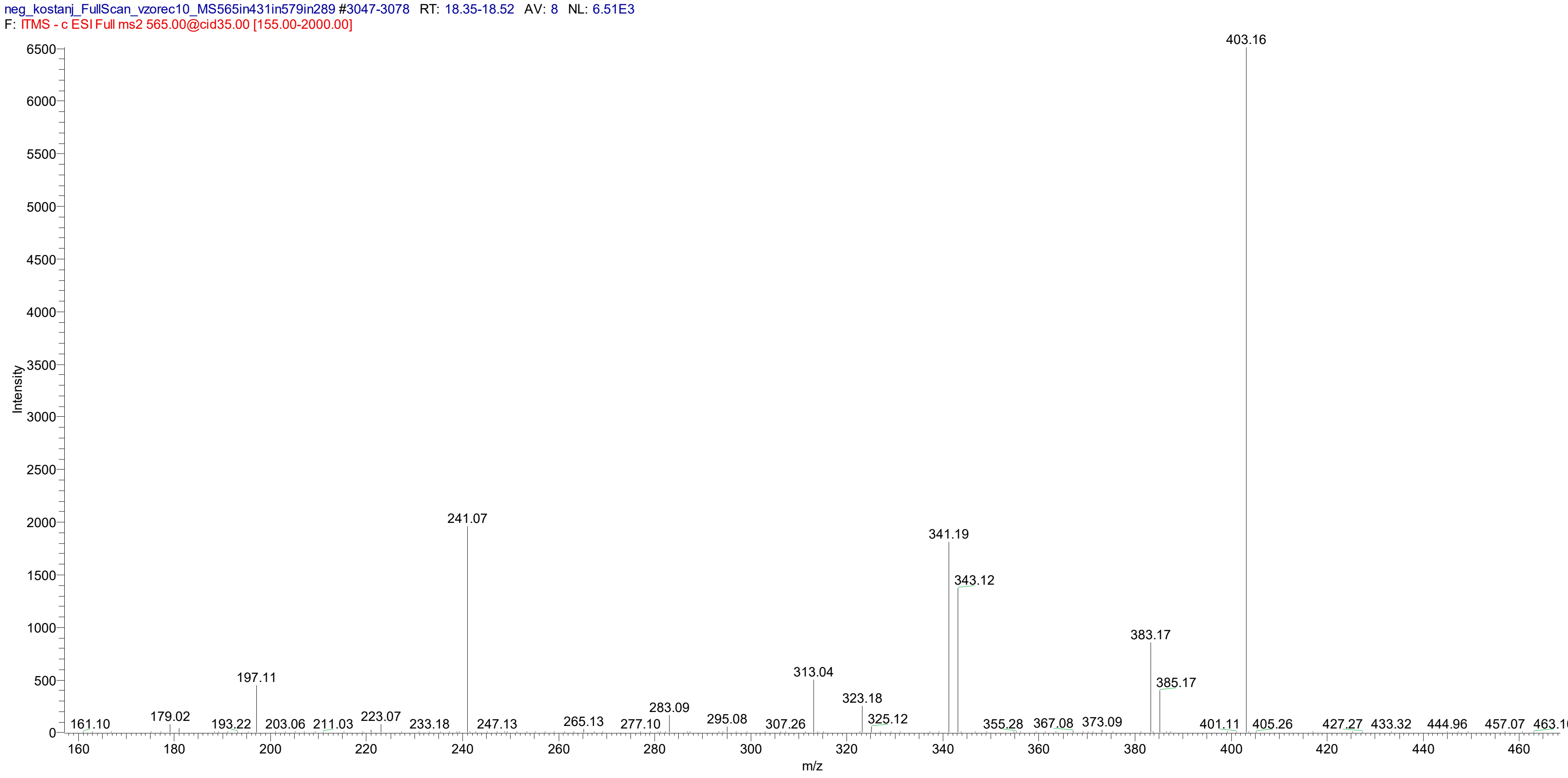

Supplement: Supplementary file 1 [file ijms-24-13086-s001.zip › ijms-2525407-supplementary.pdf]
